# rhACE2 Therapy Modifies Bleomycin-Induced Pulmonary Hypertension via Rescue of Vascular Remodeling

**DOI:** 10.3389/fphys.2018.00271

**Published:** 2018-04-09

**Authors:** Anandharajan Rathinasabapathy, Andrew J. Bryant, Toshio Suzuki, Christy Moore, Sheila Shay, Santhi Gladson, James D. West, Erica J. Carrier

**Affiliations:** ^1^Allergy, Pulmonary and Critical Care Medicine, Vanderbilt University Medical Center, Nashville, TN, United States; ^2^Pulmonary, Critical Care, and Sleep Medicine, College of Medicine, University of Florida, Gainesville, FL, United States

**Keywords:** pulmonary hypertension, pulmonary fibrosis, ACE2, bleomycin, mice

## Abstract

**Background:** Pulmonary hypertension (PH) is a progressive cardiovascular disease, characterized by endothelial and smooth muscle dysfunction and vascular remodeling, followed by right heart failure. Group III PH develops secondarily to chronic lung disease such as idiopathic pulmonary fibrosis (IPF), and both hastens and predicts mortality despite of all known pharmacological interventions. Thus, there is urgent need for development of newer treatment strategies.

**Objective:** Angiotensin converting enzyme 2 (ACE2), a member of the renin angiotensin family, is therapeutically beneficial in animal models of pulmonary vascular diseases and is currently in human clinical trials for primary PH. Although previous studies suggest that administration of ACE2 prevents PH secondary to bleomycin-induced murine IPF, it is unknown whether ACE2 can reverse or treat existing disease. Therefore, in the present study, we tested the efficacy of ACE2 in arresting the progression of group 3 PH.

**Methods:** To establish pulmonary fibrosis, we administered 0.018 U/g bleomycin 2x/week for 4 weeks in adult FVB/N mice, and sacrificed 5 weeks following the first injection. ACE2 or vehicle was administered via osmotic pump for the final 2 weeks, beginning 3 weeks after bleomycin. Echocardiography and hemodynamic assessment was performed prior to sacrifice and tissue collection.

**Results:** Administration of bleomycin significantly increased lung collagen expression, pulmonary vascular remodeling, and pulmonary arterial pressure, and led to mild right ventricular hypertrophy. Acute treatment with ACE2 significantly attenuated vascular remodeling and increased pulmonary SOD2 expression without measurable effects on pulmonary fibrosis. This was associated with nonsignificant positive effects on pulmonary arterial pressure and cardiac function.

**Conclusion:** Collectively, our findings enumerate that ACE2 treatment improved pulmonary vascular muscularization following bleomycin exposure, concomitant with increased SOD2 expression. Although it may not alter the pulmonary disease course of IPF, ACE2 could be an effective therapeutic strategy for the treatment of group 3 PH.

## Introduction

Pulmonary hypertension (PH) is a progressive, rare lung disease, which results in right heart malfunction and is fatal when untreated. The pathogenesis of PH often begins with elevated resistance in the pulmonary circulation due to endothelial dysfunction and underlying vascular remodeling, which eventually leads to increased pulmonary arterial pressure (Schermuly et al., 2011). This increased pulmonary pressure increases the right ventricular (RV) work load and maladaptive RV remodeling, and culminates in right heart failure (Schermuly et al., 2011). The median survival of a PH patient is <3 years. As per the most recent WHO guidelines, PH is classified into 5 groups, by origin: idiopathic, heritable, or drug/toxin-induced in group I, left heart disease in group 2, chronic lung disease in group 3, chronic thromboembolic pulmonary hypertension in group 4, and hematologic, systemic, or metabolic disorders in group 5 (Oudiz, 2016).

The present study focuses on chronic lung disease associated PH. The Global Burden of Disease Study on global life expectancy in 2013 (Mortality and Causes of Death, 2015) summarized that the death rate of interstitial lung disease grew more than 50% between 1990 and 2013, while its global ranking escalated from 64 (1990) to 40 (2013). Idiopathic pulmonary fibrosis (IPF) is a chronic, fatal interstitial lung disease associated with recurrent pulmonary epithelial injury and maladaptive repair, which results in a progressive interstitial fibrosis and compromised gaseous exchange (Richeldi et al., 2017). These pathological symptoms in conjunction with a concomitant endothelial dysfunction, and vascular remodeling results in group 3 PH (PH associated with IPF). PH develops in more than 32–50% of advanced IPF patients (Patel et al., 2007; Shorr et al., 2007), where it significantly impacts survival, with an increased mortality rate of 2.2–4.85-fold compared to IPF patients with no PH (Hamada et al., 2007). Despite pharmacological interventions including phosphodiesterase or endothelin receptor inhibition, or activation of prostacyclin or guanylate cyclase pathways, there has been little improvement in the mortality of IPF-associated PH (Klinger, 2016). Thus, there is great need for the development of newer therapeutic strategies to treat group 3 PH.

One promising new PH therapy is a modulator of the renin-angiotensin system (RAS). The RAS is a hormonal system of enzymes, ligands, and receptors that regulates pathophysiological events in the lung, heart, kidney, and other organs (Ferreira et al., 2010; Hsueh and Wyne, 2011; Kalra et al., 2015; Passos-Silva et al., 2015). The RAS can be categorized into two divergent axes: either the angiotensin converting enzyme (ACE), its product angiotensin II (Ang II), and the angiotensin receptor type 1 (AT1), or the angiotensin converting enzyme-2 (ACE2), the ACE2 product angiotensin-(1-7) [Ang-(1-7)], and the Ang-(1-7) receptor, Mas1 (Passos-Silva et al., 2015). Activation of the ACE2/Ang-(1-7)/Mas1 axis with administration of either rhACE2 or Ang-(1-7) also improves the cardio-pulmonary defects associated with experimental PH and PF (Shenoy et al., 2010, 2013; Meng et al., 2015). ACE2 treatment is also one of the only therapies to reverse murine BMPR2-related PAH, the cause of most heritable PH (Johnson et al., 2012), and improves right ventricular function under stress (Johnson et al., 2011).

The primary rodent model of pulmonary fibrosis and group 3 PH is a single intratracheal installation of bleomycin, which after 2 weeks recapitulates many of the functional, molecular and pathological defects associated with IPF (Mouratis and Aidinis, 2011; Meng et al., 2015), including cardiac and pulmonary vascular remodeling (Shenoy et al., 2013; Rathinasabapathy et al., 2016). A repeated low dose bleomycin insult in mice, over a period of 5 weeks, results in cardio and pulmonary vascular remodeling in addition to extensive lung fibrosis (Bryant et al., 2015; Chen et al., 2016).

The therapeutic benefits of ACE2 in prevention have been assessed in multiple bleomycin studies (Shenoy et al., 2010, 2013; Rey-Parra et al., 2012; Meng et al., 2015). These prevention studies all relied on a single dose of bleomycin, rather than repeated low doses, and the ACE2 treatment regimen was initiated either concurrent with or prior to bleomycin instillation, while development of PH/PF was acute and robust. To date, no treatment studies have assessed the therapeutic effect of ACE2 in arresting or reversing the progression of bleomycin-induced damages (treatment), over a longer course of time. Potential translation of ACE2 to human studies requires identification of potential to reverse, not just treat, IPF and IPF-associated PH, using the more human disease relevant repeated dosing model of IPF. The goal of these studies was to fill this gap in our knowledge.

## Materials and methods

### Reagents and chemicals

Bleomycin (bleomycin sulfate, Cipla, Goa, India) was purchased from the Vanderbilt University Medical Center pharmacy. Recombinant human angiotensin converting enzyme 2 (rhACE2) was a kind gift from GlaxoSmithKline, UK. α-smooth muscle actin (α-SMA), SOD2, and β-actin antibodies were purchased from Abcam (Cambridge, MA, USA) and CD68 antibody purchased from Dako (Santa Clara, CA, USA).

### Animals

All the mice utilized in this study were housed at Vanderbilt University Medical Center animal care facility, which is certified by the Association for Assessment and Accreditation of Laboratory Animal Care International. All animal protocols were approved by the Institutional Animal Care and Use Committee at Vanderbilt University Medical Center (Approval number–M12/110) in compliance with National Institute of Health guidelines. Animals housed in the conventional cages were exposed to 12:12 h, day:night cycle with free access to food (normal chow) and water. One week prior to the start of the study, animals were fed with 60% high-fat, high-calorie diet (Bio-Serv, Flemington, NJ, USA) to assist in weight maintenance, and this diet was continued until the termination of the study.

### Study design

Eighteen to 26 week old inbred male and female FVBN mice were used in this 5-week study. For the first 4 weeks, animals were challenged with bleomycin (0.018 U/g, injected i.p.), every 4 days, for a total of eight doses. Bleomycin was aseptically dissolved in saline, and the control animals for the bleomycin group were administered saline. Eighteen days following the first bleomycin injection (immediately after 6th dose), rhACE2 (1.2 mg/kg/day) was administered through mini-osmotic pumps (Durect Corporation, Cupertino, CA, USA), and the pumps were continued for 2 weeks, 1 week past the final administration of bleomycin. rhACE2 was solubilized in a buffer containing 100 mM glycine, 150 mM NaCl, 50 mM ZnCl_2_, pH 7.5. This buffer without rhACE2 was implanted in the vehicle group. Original *n* = 17 for each bleomycin group and *n* = 7 for each control group; two animals from each Bleo+vehicle and Bleo+ACE2 group died prior to obtaining invasive hemodynamics. All of these deaths occurred in the last 2 days of the study, either during or just following isoflurane anesthesia.

### Transthoracic echocardiography, hemodynamic measurements, and right ventricular hypertrophy

One day prior to the end of study, transthoracic echocardiography was performed using the Vivo 770 high-resolution image systems (VisualSonics, Toronto, Canada). On day 33, invasive hemodynamics were conducted to measure the right ventricular systolic pressure (RVSP) as described in Bryant et al. (2015). Right ventricular hypertrophy (RVH or Fulton's index) was assessed at sacrifice, and blood and tissues collected for RNA and histology. A complete blood count (CBC) analysis was performed by the Vanderbilt University Medical Center pathology core.

### Real time quantitative RT-PCR analysis

Real time RT-PCR was used to study the transcript expression of collagen type 1 (Col1), collagen type 3 (Col3), resistin like alpha (Retnla/Fizz1), mannose receptor C-type 1 (Mrc1), and arginase 1 (Arg1). In brief, total RNA was isolated from the frozen lung using RNeasy (Qiagen, Valencia, CA, USA) as per the supplier's instruction. 1 μg of total RNA was utilized to synthesis the first strand cDNA using QuantiTect reverse transcription kit (Qiagen, Valencia, CA, USA). Real-time RT-PCR was performed in a 96-well format using Power SYBR green mastermix on Applied Biosystems platform (Applied Biosystems Corporation, Foster City, CA, USA). The expression of target gene transcripts was normalized against the internal control Hprt (hypoxanthine-guanine phosphoribosyltransferase) using the comparative ΔΔCT method (2^−ΔΔ^Ct). Mouse primers are listed in Table 1.

**Table 1:** Mouse primers used for RT-PCR.

**No**	**Gene**		**Sequence**
1.	Col1	F	ACG CAT GAG CCG AAG CTA AC
		R	ACT TCA GGG ATG TCT TCT TGG C
2.	Col3	F	CCT GGA AGG GAT GGA AAC CC
		R	CAG GGC CAG TTT CTC CTC TG
3.	Retnla/Fizz1	F	CCT GCT GGG ATG ACT GCT AC
		R	CGA GTA AGC ACA GGC AGT TG
4.	Mrc1	F	AAC AAG AAT GGT GGG CAG TC
		R	TTT GCA AAG TTG GGT TCT CC
5.	Arg1	F	GAC AGG GCT CCT TTC AGG AC
		R	GCC AAG GTT AAA GCC ACT GC
6.	Hprt	F	TGC TCG AGA TGT CAT GAA GGA G
		R	TTT AAT GTA ATC CAG CAG GTC AGC

### Western blotting

A portion of the right lung lobe was homogenized in RIPA buffer with protease inhibitors for protein isolation and debris cleared by centrifugation. Proteins were resolved on a 4–12% SDS-PAGE gel (Thermo Fisher Scientific, Waltham, MA, USA), transferred to PVDF, and visualized on the ChemiDoc MP imaging system (Bio-Rad Laboratories, Hercules, CA, USA). Antibodies were diluted in 2% milk/TBST.

### Immunohistochemical analysis (IHC)

The left lung lobe was perfused with PBS, inflated with 0.8% agarose solution and stored in 10% neutral buffered formalin overnight. The following day, the fixed tissues were rinsed in 70% alcohol and subsequently processed for paraffin embedding. Paraffin-embedded tissue samples were sectioned (5 micron), de-paraffined and stained with either CD68 at 1:100, or α-SMA at 1:200. For muscularized vessel wall analysis, the total number of vessels and vessels per size group were counted at 10X (10 fields/lung; 4 animals/group). Similarly, the CD68-stained macrophages were also analyzed and counted at 10X (10 fields/lung; 4 animals/group). To analyze lung remodeling, lung sections were stained with Masson's trichrome (Sigma, St. Louis, MO, USA) and the scoring was performed in a blinded fashion as previously described (Lawson et al., 2005). 10 non-overlapping randomly photographed images were analyzed from multiple slices per animal, and the results from each animal were averaged.

### Statistics

JMP (SAS, Cary, NC) was used for statistical analysis. A two-way ANOVA with Holm-Sidak multiple comparison post-test was use to compare groups. Values are represented as mean ± SEM, and *p* ≤ 0.05 considered statistically significant.

## Results

### rhACE2 does not slow or reverse the progression of lung fibrosis in the bleomycin model of group 3 PH

**Figure 1:**
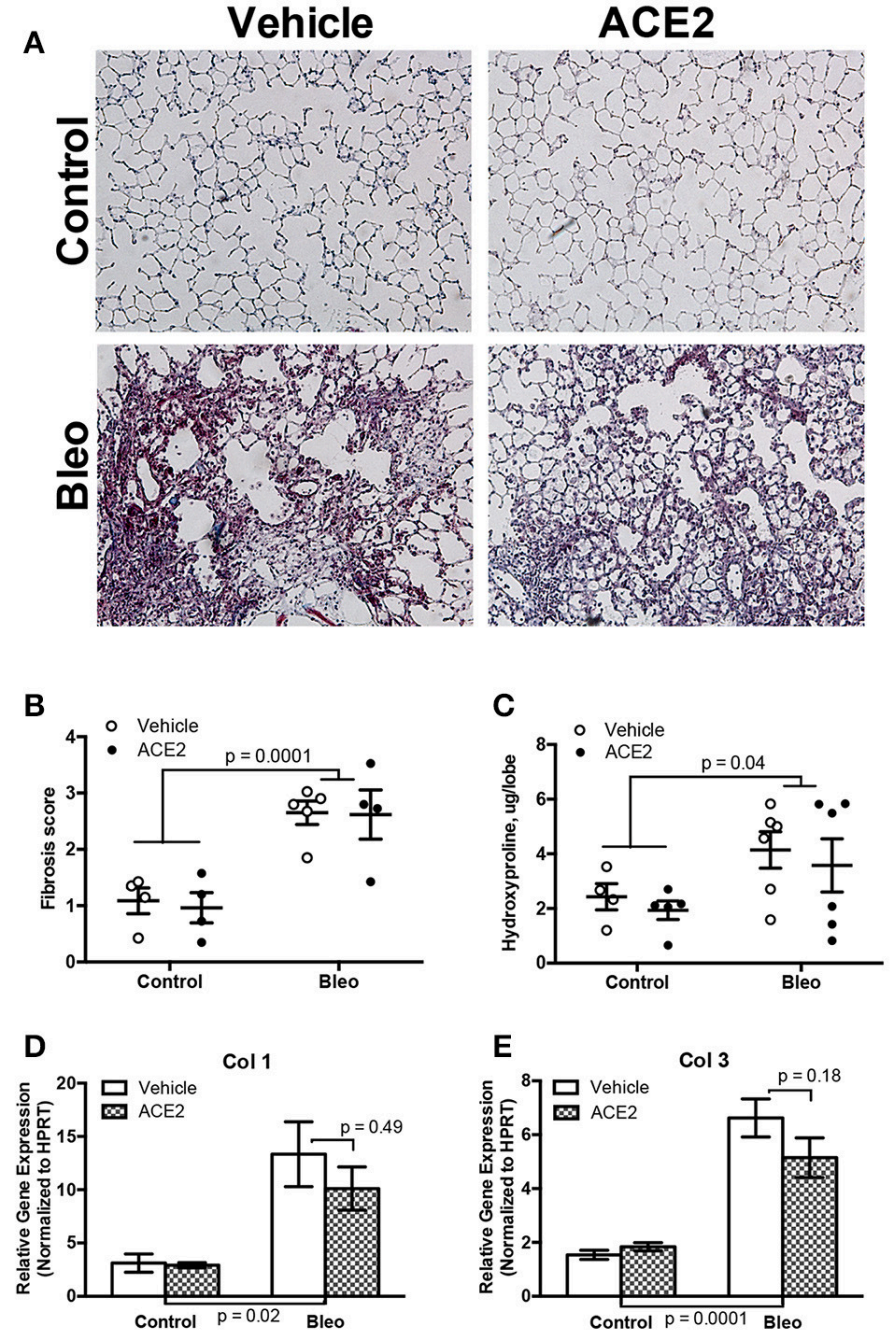
Effect of rhACE2 treatment on bleomycin-induced pulmonary fibrosis. Lung sections were stained with Masson's trichrome, visualized at 10X **(A)** and scored for parenchymal remodeling and fibrosis **(B)**. Lung collagen was estimated from tissue by hydroxyproline assay **(C)**, and expression of collagens Col1 **(D)** and Col3 **(E)** were estimated by RT-PCR. Data presented in **(D,E)** are ± SEM mean, *n* = 4, 5, 6, and 6 for the control, ACE2, Bleo and Bleo+ACE2 groups respectively. *P*-values are the result of two-way ANOVA followed by Holm-Sidak's multiple comparison post-test; in each case, bleomycin groups were significantly different from control, and ACE2-treated not significantly from vehicle.

A near-infrared blood pool imaging agent (Angiosense-750 EX, Perkin-Elmer) was used to measure pulmonary vascular leak on Day 18 after the onset of the first bleomycin injection and just prior to ACE2 pump installation. This non-invasive proxy measurement was utilized to confirm the development of pulmonary fibrosis (Supplementary Figure). Animals were then treated with ACE2 or vehicle for 2 weeks; during this time, they were exposed to bleomycin for a week and allowed a recovery period of a week before sacrifice. Administration of repeated bleomycin over the period of 4 weeks resulted in significant alteration of lung tissue architecture. Blinded scoring of Masson's trichrome-stained lung sections demonstrated a significant deposition of collagen fibers (*p* < 0.0001 by two-way ANOVA) in bleomycin-treated animals (Figures 1A,B), matched to increased hydroxyproline (Figure 1C). RT-PCR analysis demonstrated a significant increase in Col1 and Col3 expression in the lung (Figures 1D,E). Treatment of the Bleo animals with recombinant human ACE2 in weeks 4 and 5 had no significant effect on histology (*p* = 0.62, Figures 1A,B), hydroxyproline (Figure 1C), or expression of Col1 and Col3 (Figures 1D,E), compared with vehicle-treated animals.

### RV dynamics and cardiac function associated with group 3 PH animals

**Figure 2:**
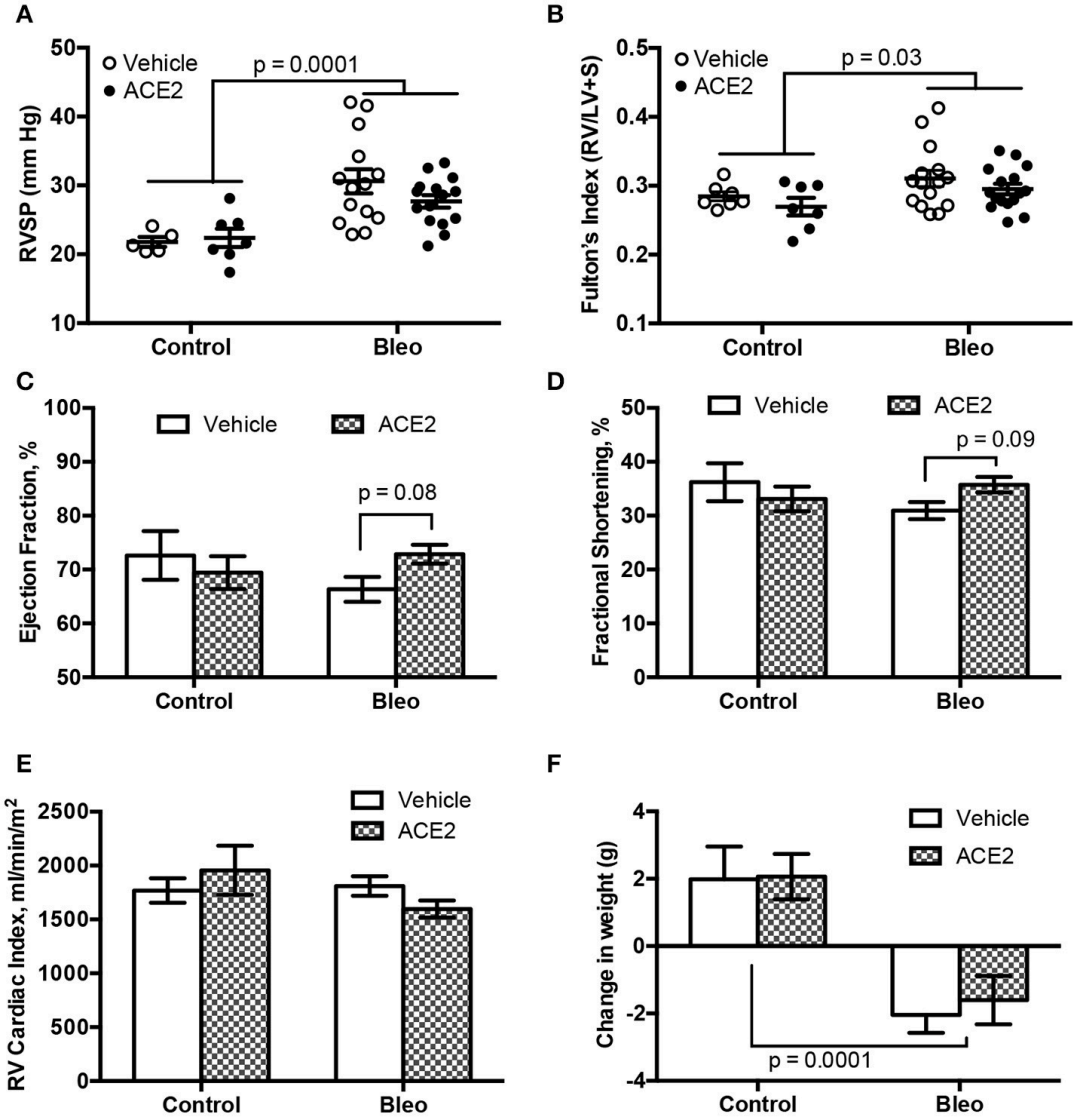
RVSP and cardiac function in group 3 PH animals. RVSP, measured by RV catheterization, and hypertrophy, measured by Fulton's index, both increase with bleomycin treatment **(A,B)**. ACE2 significantly improved the fractional shortening and ejection fraction in bleomycin-treated animals, compared with vehicle **(C,D)**, while there was no change in RV cardiac output normalized for weight across groups **(E)**. ACE2 did not significantly decrease the weight loss associated with Bleo treatment **(F)**. Data are mean ± SEM; *n* = 7, 7, 15, and 16 for the control, ACE2, Bleo and Bleo+ACE2 groups respectively in **(B–F)**, and *n* = 5, 7, 14, and 15 in **(A)**. Comparisons shown are the result of two-way ANOVA followed by Holm-Sidak's post-test.

As expected (Figures 2A,B), bleomycin insult resulted a significant increase in RVSP (Con; 21.8 ± 0.7 *vs*. Bleo; 30.6 ± 1.7, mmHg, *p* < 0.0001 by two-way ANOVA) with a concomitant change in Fulton's index (Con; 0.28 ± 0.006 *vs*. Bleo; 0.31 ± 0.01, *p* = 0.03). ACE2 reduced the RVSP variance (*p* = 0.028 by *F*-test) but failed to overall significantly decrease RVSP in response to bleomycin (*p* = 0.19 by two-way ANOVA/Holm-Sidak). We performed transthoracic echocardiography (echo) to determine cardiac function in these animals. Bleomycin-treated animals showed only a trend toward decreased LV ejection fraction and fractional shortening, which were reversed with ACE2 treatment (Figures 2C,D). There was no overall change in cardiac index with bleomycin (Figure 2E), and so ACE2 had no impact. Cardiac index (cardiac output normalized to body surface area) rather than cardiac output is displayed because of the large differences in weight between control and bleomycin treated animals (Figure 2F), also not impacted by ACE2 treatment.

### rhACE2 does not alter lung inflammation in group 3 PH animals

Because remodeling is often caused by inflammation, we assessed inflammatory cells in the lungs and circulation. In the peripheral circulation, bleomycin slightly increased white blood count, circulating neutrophil numbers, red cell distribution width, and platelet count (Table 2). ACE2 treatment had no impact on any of these parameters, but did cause a slight increase in mean platelet volume, likely not functionally meaningful.

**Table 2:** Results of complete blood count.

	**Control**		**Bleomycin-treated**		**Significance**	
	**Vehicle**	**ACE2**	**Vehicle**	**ACE2**	**Bleo:**	**ACE2:**
White blood count (10^9^/L)	1.7	2.2	2.6	2.5	0.037	
Neutrophil # (10^9^/L)	0.6	0.9	1.2	1.2	0.009	
Lymphocyte # (10^9^/L)	1.1	1.2	1.3	1.2		
Monocyte # (10^9^/L)	0.08	0.10	0.10	0.12		
Hematocrit (%)	49.3	52.2	51.8	54.1		
Red cell distribution width (%)	13.8	13.9	14.2	14.3	0.015	
Platelet count (10^9^/L)	837.1	717.7	1005.6	941.9	0.013	
Mean platelet volume (fL)	5.23	5.37	5.33	5.39		0.027

*Data are mean ± SEM, n = 7, 7, 14, and 15 for the control, ACE2, Bleo and Bleo+ACE2 groups respectively; comparisons between groups were made with two-way ANOVA followed by Holm-Sidak's multiple comparison post-test*.

**Figure 3:**
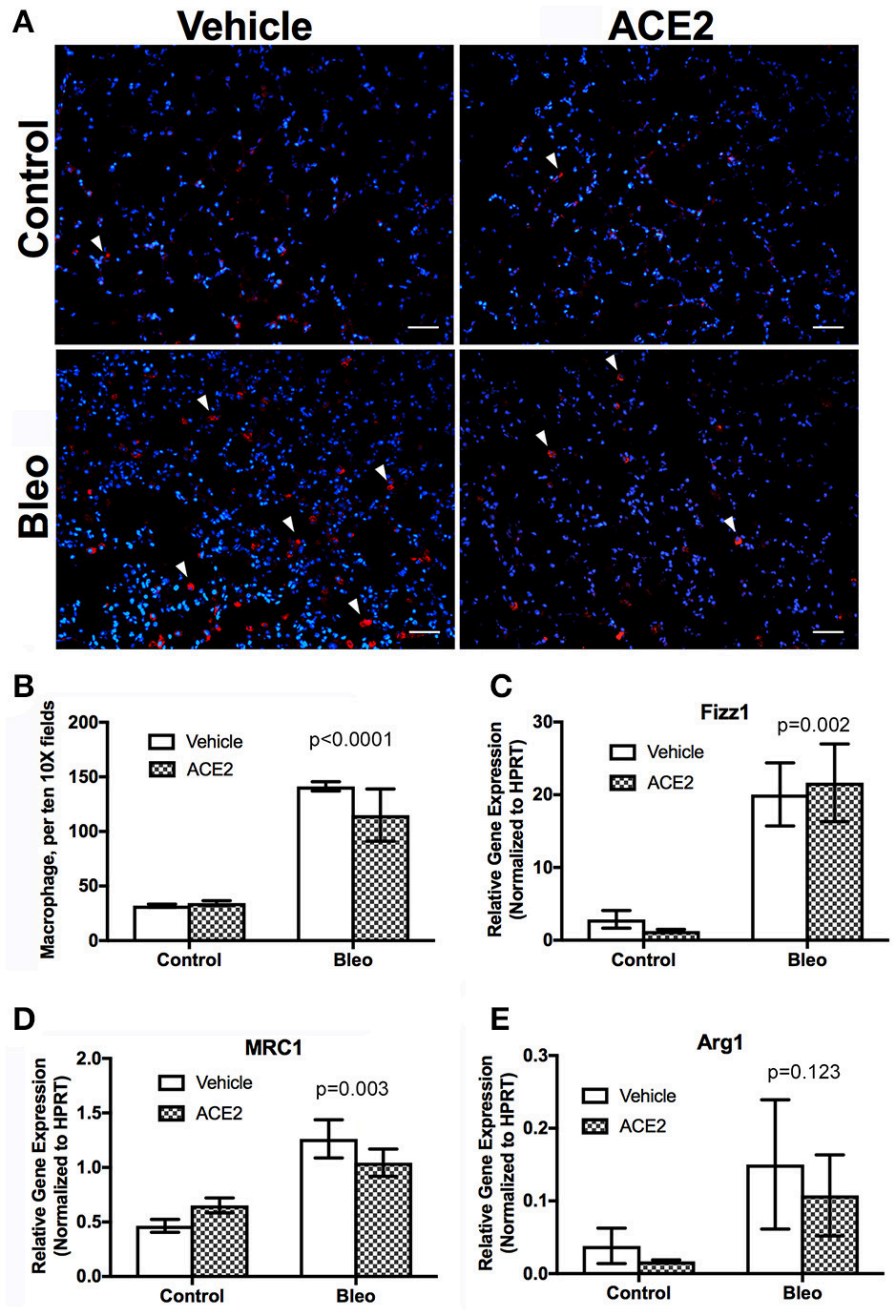
Pulmonary macrophage dynamics in group 3 PH animals. Lung sections were stained for CD68 and random fields photographed at 10X and CD68^+^ cells counted **(A,B**, *n* = 4). RNA was isolated from frozen lungs and RT-PCR used to assess the expression of markers of macrophage polarization. **(C)** Retnla (Fizz1), **(D)** MRC1, **(E)** Arg1. Data presented in **(C–E)** are mean ± SEM, *n* = 4, 5, 6, and 6 for the control, ACE2, Bleo and Bleo+ACE2 groups respectively. Bleomycin-treated groups significantly differed from control groups by two-way ANOVA followed by Holm-Sidak's post-test, *p* < 0.0001 in **(B–E)**. Scale bars shown are 50 microns.

To assess the status of inflammatory cells in the lung, we stained sections for CD68, a marker for monocytes/macrophages, and found that macrophage presence was significantly increased in the Bleo lungs (Figures 3A,B), with an approximately 4-fold increase in pulmonary macrophages. Because IPF patients predominantly have M2 macrophage lung infiltrates (He et al., 2013), we used RT-PCR to more closely examine macrophage polarization following bleomycin administration. Expression of the M2 macrophage markers Retnla (Fizz1), Mrc1, and Arg1 were all significantly elevated in the Bleo lung (Figures 3C–E), with Fizz1 increased 10-fold (greater than the increase in macrophages). However, ACE2 did not reduce this increase in M2 markers.

### rhACE2 treatment improves the pulmonary vascular remodeling in group 3 PH animals

**Figure 4:**
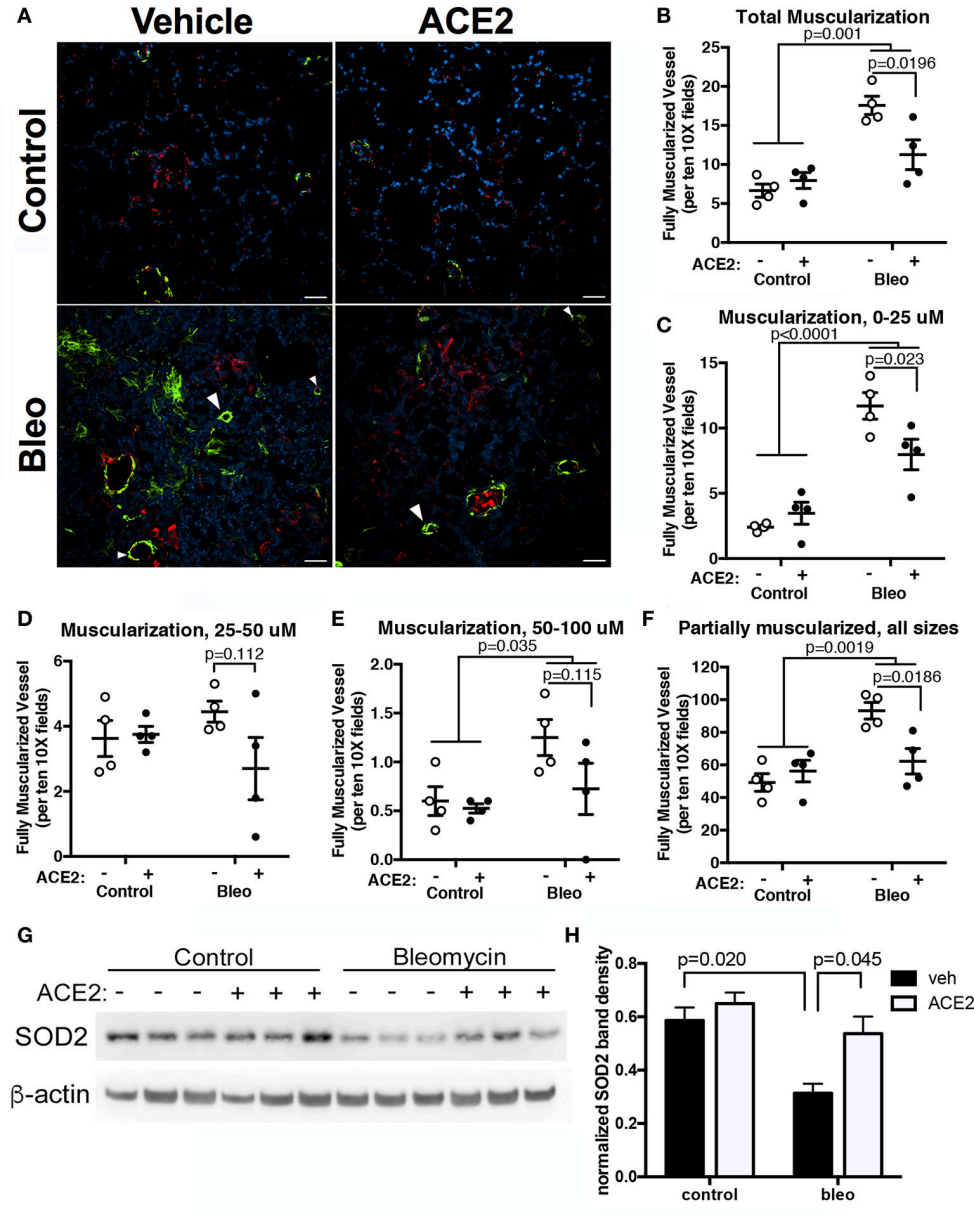
rhACE2 treatment improves the pulmonary vascular remodeling in group 3 PH animals, while increasing pulmonary SOD2 expression. Lung sections were stained for α-SMA and von Willebrand factor, and random fields photographed at 10X. **(A)** Representative images of α-SMA-positive muscularized vessels (arrowheads). **(B–E)** ACE2 treatment decreased per field complete vessel muscularization regardless of vessel size: total (B), 0–25 μm **(C)**, 25–50 μm **(D)**, and 50–100 μm **(E)**, as well as partial muscularization of all vessels (**F**; mean ± SEM, *n* = 4 for all experimental groups). The results of two-way ANOVA followed by Holm-Sidak's post-test are shown; scale bars are 50 microns. **(G–H)** Bleomycin decreased SOD2 expression in mouse lung, which was normalized in mice treated with ACE2. Bands were quantified in ImageJ and normalized to β-actin. Shown are the results of a two-way ANOVA comparison followed by Holm-Sidek test (*n* = 3).

To assess pulmonary vascular remodeling, we stained lung sections with α-SMA. Scoring of non-overlapping images at 10x demonstrated a significant muscularization of pulmonary vessel wall in the Bleo animals (Figures 4A–E). A vessel was considered to have total muscularization when ≥70% of the vessel perimeter stained positive for α-SMA. Muscularization was further characterized based on vessel diameter. There was pronounced remodeling in the smaller (*p* = 0.0001, Figure 4C) and larger vessels (*p* = 0.03, Figure 4E) following Bleo administration. In agreement with the trends in RVSP and cardiac function, rhACE2 treatment attenuated muscularization (Figures 4A–E), in Bleo animals, particularly in vessels <25 microns in size (Figure 4C). Similarly, mice treated with ACE2 had fewer vessels of any size that were partially (<70%) muscularized (Figure 4F). We have recently investigated the effects of short-term ACE2 infusion in human PAH patients, and found reduced oxidant stress and increased plasma superoxide dismutase-2 (SOD2) with ACE2; in parallel, we have found that application of a synthetic agonist for Mas receptor increases SOD2 expression in porcine pulmonary vessels (Carrier et al., 2016). Because Mas receptor activation or ACE2 treatment can increase SOD2 protein levels, and SOD2 has been shown to decrease vascular smooth muscle proliferation and neointimal formation, we examined the effects of ACE2 on pulmonary SOD2 expression in Bleo-treated mice. Bleomycin instillation reduced SOD2 expression, but was normalized in Bleo animals treated with ACE2 (Figures 4G,H). Taken in combination, these data suggest that the significant reduction in vessel muscularization with ACE2 in bleomycin treated mice was not the result of altered inflammatory cell numbers but possibly due to improved SOD2 expression.

## Discussion

Although the body of existing literature suggests a beneficial effect of ACE2 in various animal models of PH and PF (Shenoy et al., 2010, 2013; Meng et al., 2015), our study would be the first to examine recombinant human ACE2 as treatment for previously established pulmonary fibrosis, and against the development of group 3 PH and the associated cardiac abnormalities in animals following extended Bleo exposure. Our data suggest that rhACE2 treatment does not significantly alter PF in these animals, but prevents the vascular remodeling that may lead to PH, and improves some markers of cardiac function.

Although the onset of fibrotic scar in the IPF lung is idiopathic, this chronic lung disease is promoted by repeated parenchymal injury, vascular leak, recruitment and differentiation of fibroblasts, and pathologic remodeling in pulmonary circulation that culminates in *cor pulmonale* (Fernandez and Eickelberg, 2012). Bleomycin insult in rodents recreates these cascades of events with a significant disruption in the expression pattern of RAS components (Meng et al., 2015). A single injection of bleomycin in rodents causes the robust development of lung fibrosis and symptoms of PH, including elevated RVSP and RV dysfunction (Shenoy et al., 2010, 2013). To better mimic the slower progression of group 3 PH, in this study we utilized an animal model with repeated Bleo insult. The advantage of repeated Bleo administration is that it better mimics the sustained alveolar epithelial injury of IPF (Fernandez and Eickelberg, 2012), and pulmonary recovery, which limits the usefulness of the single-administration mouse model, is prevented. Functionally, these animals exhibited severe alteration in the lung architecture in addition to cardio-pulmonary vascular remodeling and elevated pulmonary arterial pressure. Recently, Bryant et al. demonstrated that vascular leak and interaction between vascular smooth muscle and endothelial cells in animals given repeated bleomycin results in vascular remodeling, independent of the severity of PF (Bryant et al., 2016). Clinically, IPF-PH results in a progressive, irreversible lung damage; therefore, the treatment regimen for those patients are aimed at improving the pulmonary vascular rather than the fibrotic symptoms (Klinger, 2016). One weakness of the bleomycin rodent IPF model is that it depends on an initial inflammatory reaction, and compounds that prevent this inflammatory phase can show false promise for IPF treatment (Moeller et al., 2008). Thus, although multiple studies describe the preventive effect of ACE2 and Ang-(1-7) in bleomycin-induced PF, for better translation to human disease it is imperative to assess post-induction treatment. Here, we demonstrate that rhACE2 treatment after induction of IPF with repeated Bleo does not affect pulmonary fibrogenesis, collagen expression, and extracellular remodeling, yet still decreases vascular remodeling. Similar to previous studies from our lab, using different models of pulmonary arterial hypertension (West et al., 2016a,b), the modest increase in RVSP was not enough to cause cardiac dysfunction as measured by echo. Thus, we are unable to conclusively comment on any rescue effects of ACE2 on RV function in this model. However, our observations are in line with our findings that rhACE2 treatment primarily improved the PH symptoms in the present study.

The RAS is dysregulated in IPF, animal models of PF, PH, and in the animal PH model of RV pressure overload (Johnson et al., 2011; Shenoy et al., 2013; Meng et al., 2015). In IPF and bleomycin lungs, there is attenuated expression and activity of ACE2 (Li et al., 2008). Similarly, a number of groups have demonstrated the dominant expression pattern of ACE and AT_1_ receptor over ACE2 and Mas1 receptor in pulmonary and cardiovascular diseases (Qi et al., 2011; Shenoy et al., 2013; Meng et al., 2015). Activation of the ACE2/Ang-(1-7)/Mas1 axis by the administration of ACE2 or Ang-(1-7) results in enhanced ACE2 activity and promotes expression of ACE2/Mas1 over ACE/AT_1_ in PF and PH; furthermore, augmentation of the ACE2/Ang-(1-7)/Mas1 axis improved the pathological symptoms associated with these heart and lung tissues (Shenoy et al., 2013; Meng et al., 2014, 2015). While ACE2 can also hydrolyze AngI to produce the bioactive peptide Ang-(1-9), its affinity for AngII is approximately 400x greater (Vickers et al., 2002); thus, the effects seen with ACE2 administration are more likely to be mediated by Ang-(1-7). Our group recently conducted an open-label phase IIa pilot study of rhACE2, and found that even an acute 4 h infusion of rhACE2 greatly improved the Ang II/Ang-(1-7) ratio, with a concomitant increase in the cardiac output (Hemnes et al., 2017). In the present study, we used continuously infused rhACE2 to activate the ACE2/Ang-(1-7)/Mas1 axis following induction of PF in mice with repeated Bleo injection. Although we did not quantify the expression pattern or the peptide levels of RAS members pre- and post-rhACE2 treatment, we have previously found that administration of rhACE2 for 2 weeks increases RV Mas1 expression in a mouse model of PAH (Johnson et al., 2011). To support the involvement of the Ang-(1-7)/Mas1 axis in this study, we have found that ACE2 treatment normalized pulmonary expression of SOD2 in Bleo mice; this complements our previous finding that synthetic agonist of the Mas receptor increases SOD2 expression in porcine pulmonary vessels (Carrier et al., 2016).

In our study, ACE2 treatment reduced muscularization of pulmonary vessels following bleomycin. We also observed an extensive lung tissue remodeling in conjunction with an accumulation of macrophages after repeated bleomycin. Numerous studies on PH have implicated that inflammatory signals produced at the site of injury due to infiltration of monocytes, macrophages, dendritic or natural killer cells or any other immune cells compliment disease progression by directly promoting pulmonary vascular remodeling (El Chami and Hassoun, 2012; Rabinovitch et al., 2014). In this study, the increased CD68^+^ macrophages and expression of M2 macrophage markers in the bleomycin lung were not significantly diminished with ACE2 treatment, suggesting that reduced macrophage infiltration was not responsible for the reduction in vessel muscularization with ACE2 in bleomycin treated mice. Ang-(1-7) does reduce vascular smooth muscle proliferation *in vitro* (Freeman et al., 1996), and following vascular injury (Strawn et al., 1999) suggesting a possible direct effect on vascular smooth muscle. Short-term ACE2 treatment increases SOD2 expression in PH patients (Carrier et al., 2016), and SOD2 overexpression decreases proliferation and migration of vascular SMC while normalizing apoptosis (Archer et al., 2010; Wang et al., 2012). Because SOD2 is known to be reduced in PH patients (Bowers et al., 2004; Archer et al., 2010), and SOD2 siRNA creates a PAH phenotype in vascular SMC (Archer et al., 2010), this suggests that ACE2 treatment could fill a necessary void in PH treatment by decreasing vascular remodeling.

Noticeable shortcomings of our study are the modest effects of bleomycin-induced PH on cardiac function, and the variable and limited rescue of PF and PH symptoms by rhACE2. The length of our rhACE2 treatment, and thus the study length, was limited by the potential immune response of mice to a recombinant human protein. Therefore, a follow-up study that used murine ACE2 to extend bleomycin exposure and mACE2 treatment time may find more significant effects both of Bleo-induced PF as well as mACE2 rescue and strengthen the case for the therapeutic benefits of rhACE2 in group 3 PH. However, our investigation is the first attempt to demonstrate that the rhACE2 limited the progression of group 3 PH in animal model, by improving vascular remodeling and normalizing SOD2 expression. Limiting vascular muscularization and possibly improving RV function would dramatically improve patient quality of life, since the available pharmacologically agents marginally benefit the irreversible chronic lung conditions in group 3 PH, and one study of lungs from 62 mixed-origin PH patients demonstrated that prostacyclin treatment does not reduce the pathology of vascular remodeling (Stacher et al., 2012). Although triple-combination therapy including synthetic prostacyclin may reduce pulmonary arterial pressure in group 1 PAH patients (Sitbon et al., 2014), it is yet unknown whether this will benefit IPF-associated PAH though the TRITON clinical trial investigating the efficacy of triple-combination therapy is currently including group 3 PAH patients (clinicaltrials.gov study NCT02558231). Despite the limitations of this investigation, the outcome of rhACE2 treatment in bleomycin animals contributes to a growing body of evidence that warrants further consideration of rhACE2 or promoters of the ACE2/Ang-(1-7)/Mas axis for treatment of group 3 PH.

## Author contributions

AR, AB, JW, and EC conceived and designed the experiment. AR, AB, EC, TS, CM, SS, and SG performed the experiment. AR, JW, and EC analyzed the data. AR, JW, and EC wrote the paper.

### Conflict of interest statement

The authors declare that the research was conducted in the absence of any commercial or financial relationships that could be construed as a potential conflict of interest.

## Abbreviations

α-SMA: alpha smooth muscle actin
ACE: angiotensin converting enzyme
Arg1: arginase 1
AT1: angiotensin type 1 receptor
Bleo: bleomycin
Col1: collagen, type 1
Col3: collagen, type 3
echo: echocardiography
EF: ejection fraction
IPF: idiopathic pulmonary fibrosis
LV: left ventricle
Mas1: Mas receptor
MRC1: mannose receptor C-type 1
PH: pulmonary hypertension
rhACE2: recombinant human angiotensin converting enzyme 2
RAS: renin angiotensin system
Retnla: Resistin like alpha
RV: right ventricle
RVH: right ventricular hypertrophy
RVSP: right ventricular systolic pressure
SOD2-: superoxide dismutase-2 (manganese superoxide dismutase)
WBC: white blood cell.
