# Supplementary material for: rhACE2 Therapy Modifies Bleomycin-Induced Pulmonary Hypertension via Rescue of Vascular Remodeling
Source: Front Physiol. 2018 Apr 9;9:271. doi: 10.3389/fphys.2018.00271 (PMC5922219; doi:10.3389/fphys.2018.00271)

Supplementary Figure:

Following 3 weeks of bleomycin treatment and just prior to osmotic pump implementation, mice underwent near-infrared spectroscopic imaging to determine degree of vascular permeability in pulmonary tissue. In brief, mice underwent 4 µL/g retro-orbital injection of a 2 mmol solution of AngioSense 750 EX (Perkin Elmer, Waltham, MA) in PBS 1 day prior to imaging, and were prepared for imaging by having the chest and abdomen shaved. The following day, lung fluorescent signal was captured on a Pearl small-animal imager (LI-COR, Lincoln, NE) and quantified with normalization to the mouse bladder infrared signal intensity. This process was repeated two days prior to sacrifice, to estimate leakage after ACE2 or vehicle treatment. Shown are images of a control (A) and bleomycin-treated (B) mouse, with the target areas of quantification outlined. The intensity of infrared signal in the pulmonary region normalized to that of the nose, or "pulmonary leak" is shown in control and bleomycin-treated mice prior to (C) and following (D) ACE2 or vehicle administration. Comparisons shown are by unpaired t-test with Welsh's correction (C) or by two-way ANOVA followed by Holm-Sidak's multiple correction post-test (D).

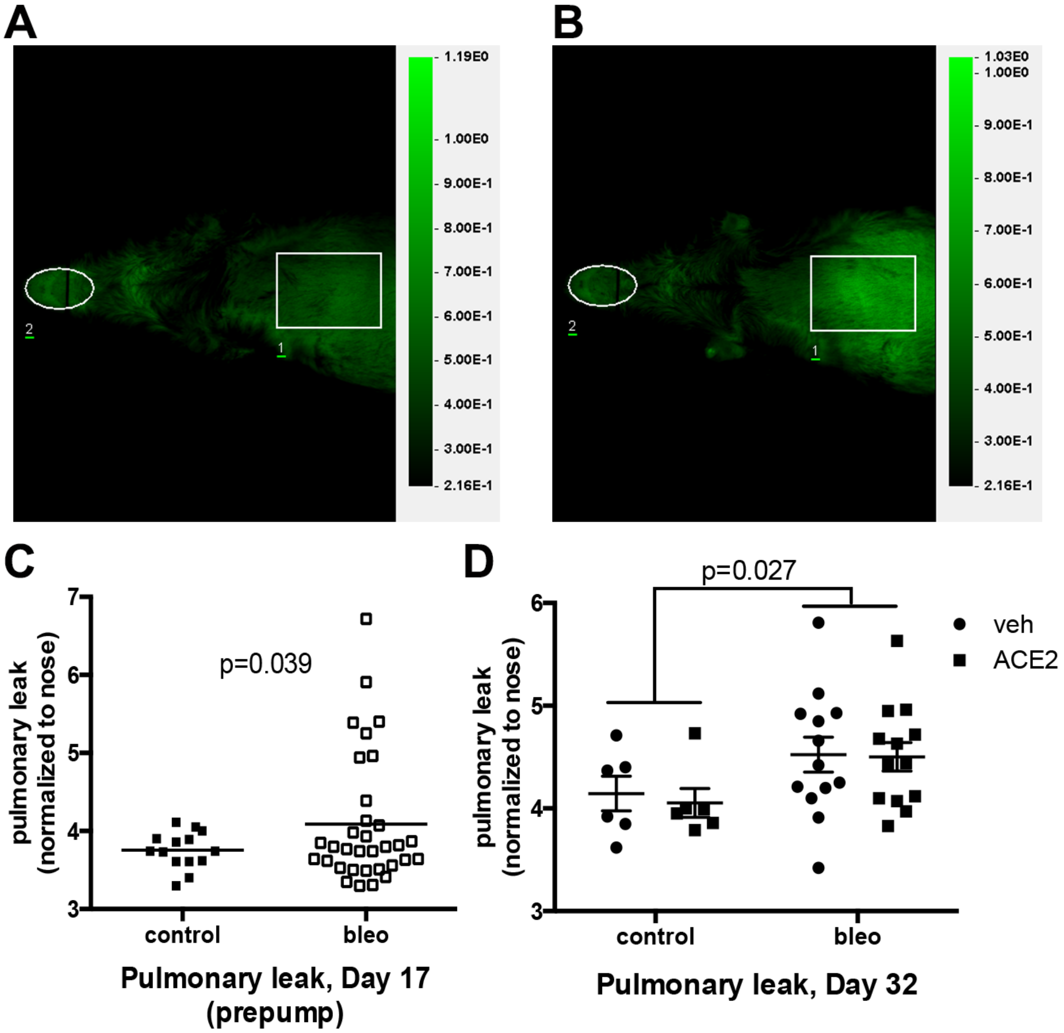

Supplement: Supplementary file 1 [file DataSheet1.pdf]
